# Modeling the Potential Distribution of *Bacillus anthracis* under Multiple Climate Change Scenarios for Kazakhstan

**DOI:** 10.1371/journal.pone.0009596

**Published:** 2010-03-09

**Authors:** Timothy Andrew Joyner, Larissa Lukhnova, Yerlan Pazilov, Gulnara Temiralyeva, Martin E. Hugh-Jones, Alim Aikimbayev, Jason K. Blackburn

**Affiliations:** 1 Emerging Pathogens Institute and the Department of Geography, University of Florida, Gainesville, Florida, United States of America; 2 Kazakh Science Center for Quarantine and Zoonotic Diseases, Almaty, Kazakhstan; 3 Department of Environmental Science, School of the Coast and Environment, Louisiana State University, Baton Rouge, Louisiana, United States of America; University of Liverpool, United Kingdom

## Abstract

Anthrax, caused by the bacterium *Bacillus anthracis,* is a zoonotic disease that persists throughout much of the world in livestock, wildlife, and secondarily infects humans. This is true across much of Central Asia, and particularly the Steppe region, including Kazakhstan. This study employed the Genetic Algorithm for Rule-set Prediction (GARP) to model the current and future geographic distribution of *Bacillus anthracis* in Kazakhstan based on the A2 and B2 IPCC SRES climate change scenarios using a 5-variable data set at 55 km^2^ and 8 km^2^ and a 6-variable BioClim data set at 8 km^2^. Future models suggest large areas predicted under current conditions may be reduced by 2050 with the A2 model predicting ∼14–16% loss across the three spatial resolutions. There was greater variability in the B2 models across scenarios predicting ∼15% loss at 55 km^2^, ∼34% loss at 8 km^2^, and ∼30% loss with the BioClim variables. Only very small areas of habitat expansion into new areas were predicted by either A2 or B2 in any models. Greater areas of habitat loss are predicted in the southern regions of Kazakhstan by A2 and B2 models, while moderate habitat loss is also predicted in the northern regions by either B2 model at 8 km^2^. Anthrax disease control relies mainly on livestock vaccination and proper carcass disposal, both of which require adequate surveillance. In many situations, including that of Kazakhstan, vaccine resources are limited, and understanding the geographic distribution of the organism, in tandem with current data on livestock population dynamics, can aid in properly allocating doses. While speculative, contemplating future changes in livestock distributions and *B. anthracis* spore promoting environments can be useful for establishing future surveillance priorities. This study may also have broader applications to global public health surveillance relating to other diseases in addition to *B. anthracis*.

## Introduction

*Bacillus anthracis* is a spore-forming bacterium that is endemic to specific soil environments and the causative organism for anthrax, an infectious disease primarily found in herbivorous wildlife and livestock species, and secondarily in humans [1]. Limited data are available to define the geographic extent of environmental variables that support long-term *B. anthracis* spore survival, but current literature suggests that *B. anthracis* likely replicates in the animal host and can then survive for long periods in specific soil environments [2]–[5]. However, new evidence on the potential role of bacteriophages and soil-dwelling invertebrates (e.g. worms) suggests a more complicated life cycle for *B. anthracis* in soil that may or may not require a mammalian host for multiplication and may provide an alternative to a spore-only survival mechanism in soil [6]. In either case, it is plausible that these scenarios require similar soil conditions to those described for “spore survival” in the earlier literature. Hugh-Jones and Blackburn [7] summarize the general soil conditions for *B. anthracis* survival from a large body of literature as humus-rich, alkaline soils with pH >6.0 and distributed across the steppe and grassland soils.

Until recently, knowledge concerning the distribution of these environments was limited to studies that focused primarily on the distribution of *B. anthracis* in North America[1], [8], [9] and parts of Africa [5], but a recent study in Kazakhstan revealed some of the environmental constraints of *B. anthracis* on the landscape (Aikembayev unpublished manuscript). A second study [10] confirmed that the majority of anthrax cases in Kazakhstan over the last century affected large (cattle) and small ruminants (sheep and goats). It has also been determined that human anthrax cases in Kazakhstan are primarily caused by exposure to infected animals – usually cattle, sheep, horses, or goats [11]. Anthrax cases were predominantly cutaneous infections and were most often linked directly to the slaughtering and/or butchering of infected animals and no reports of human to human transmission occurred in the study. People in rural environments were more commonly infected because of a lifestyle that was more involved with livestock management/production and insufficient vaccination efforts (lack of access, availability, surveillance, etc.) were the main reason for infection in Kazakhstan and the surrounding central Asian countries [11]. Since exposure to livestock is a major source of anthrax infections in humans, it is also important to consider the factors that help to regulate domestic livestock numbers. One such study [12] examined factors that regulated domestic livestock numbers over the past century in Kazakhstan and determined that the timing and amount of precipitation are the most crucial factors.

Recent studies have attempted to better understand the geographic distribution of *B. anthracis* and anthrax outbreaks in Kazakhstan by employing GIS, spatial analysis, and molecular genotyping techniques [10] and spatial statistics and ecological niche modeling (Aikembayev unpublished manuscript). Ecological niche modeling has often been used to model a species' ecological and geographic distribution. Many different ENM approaches have been utilized for various studies including the presence-absence approach and the presence-only modeling approach [13]. The presence-absence modeling approach requires that presence and absence locality data be provided in order to model the ecological niche of a species. Absence data, however, are often difficult to validate because many areas that may be classified as being absent of a certain species may, in actuality, provide a suitable habitat [14]. In some situations, a species may not have been observed in an area where it actually does exist. For example, sampling gear biases may limit the successful capture of live specimens [15], [16] or sampling efforts may not exhaustively search all possible areas within the species' range. In the case of pathogen-based studies, proper diagnostics, test sensitivity, and detection thresholds must all be considered when defining the causative agent as present or absent.

The presence-only modeling approach requires locality data to create a predicted geographic distribution of a species based on environmental parameters that exist where the species is confirmed to be present [14]. Pseudo-absence data are often generated in this approach to determine areas that do not match the environmental parameters of areas that are known to be present for a particular species [14]. The presence-only ENM approach has been successfully employed to model the potential geographic distribution of a number of taxa [17]–[21], including disease vectors [22]–[27] and disease organisms [8], [28]. An ENM constructs a definition of the niche of an individual species in ecological (variable) space and predicts its potential geographic distribution through the analysis of relationships between combinations of environmental variables (e.g., temperature, precipitation, and elevation derived from digital maps or satellite data) and species' locality data [8].

The ecological niche can be defined as those environmental conditions that allow a species to maintain its population without immigration [29], [30]. That definition was later expanded to state that the presence of a species is correlated to quantifiable environmental and biotic variables that promote its survival, or a region in multi-dimensional space that describes states of the environmental variables which are suitable for the species to exist (i.e. a hypervolume of parameters) [31]. The complexity of intra- and inter-specific interactions was recognized and niche space was consequently sub-divided into a fundamental niche (maximum extent of environment that can sustain its population) and a realized niche (actual environment that a species inhabits). Theoretically, a species often cannot inhabit its entire fundamental niche because of disturbance (e.g, habitat fragmentation) [17], inter-specific competition [32], or intra-specific limits (e.g. vagility, reproductive success) [33].

An ENM known as the Genetic Algorithm for Rule-set Prediction (GARP), that can be broadly defined as a fundamental niche modeling approach [34], was recently used to examine the geographic distribution of *B. anthracis* in the United States (US) under current [8] and future ecological conditions [35]. Another study from Kazakhstan also used GARP to model the potential geographic distribution of environments that likely support long-term persistence of *B. anthracis* and confirmed that repeat livestock anthrax epizootics occur within that predicted geographic range of the organism (Aikembayev unpublished manuscript). In that study it was predicted that the northern and southeastern regions of Kazakhstan may provide a suitable habitat for *B. anthracis* survival, while the interior and western regions of the country are potentially unsuitable for *B. anthracis*.

Recent work has advocated for the use of ENM as a method to provide improved surveillance strategies for anthrax across the United States [8]. The same is true for Kazakhstan. The geographic potential of *B. anthracis* covers a very large area in both countries, but vaccination in both cases is usually administered as a reactionary measure in response to outbreaks. However, knowledge of the distribution of *B. anthracis* can allow for better monitoring and control measures in areas where the disease (or its causative agent) is predicted to be present [8]. The use of ENM to model the current distribution of *B. anthracis* in Kazakhstan also produced similar results intended to improve surveillance and target control strategies in an effort to be more proactive in the management of anthrax outbreaks in livestock (Aikembayev unpublished manuscript).

A major advantage of GARP (and other ENMs) is the ability to project the future distribution of a species based on its current relationship to environmental variables and the prediction of climate change that will occur over the geographical area inhabited by the species. The theory of ecological niche conservatism with respect to ENM helps to support this approach [36]. It states that a species maintains the same ecological niche over very long periods of time. This allows for the prediction of habitat change for a species based on future climate change scenarios [17], [37]–[43]. However, some uncertainty surrounds the prediction of a species' future distribution [39], [42]–[44]. It has been argued appropriately that we have no means of determining the changing interactions between species because of climate change [44]. However, Global Climate Models (GCMs) do provide some measures of confidence and intensive speculation through the use of the best available current and future bioclimatic data may help to plan for possible future changes in a species' distribution.

Since the release of future climate/emissions scenarios by the Intergovernmental Panel on Climate Change (IPCC) [45], many published studies have predicted future climate change patterns that may occur in central Asia over the next 50–100 years [46]–[49]. Multiple studies have concluded that 1) an increase in annual precipitation over most of Asia with 2) an overall rise in temperatures that is most pronounced in the winter months has occurred over the past several decades [50] and may continue to occur in the future [48], [51]. Annual, inter-annual, and decadal trends have also been studied recently to analyze the relationship between atmospheric forcing mechanisms (e.g., teleconnections) and recent Eurasian climate variability [52], [53]. The importance of snow cover extent changes and its possible role as an amplifier of regional atmospheric patterns has also been examined [53]. Snow season lengths, snow depths, and annual snow accumulation variability have also been studied in coordination with global sea surface temperature (SST) variability, regional atmospheric changes (increased precipitation and increased temperatures overall), and regional atmospheric oscillation patterns over varying periods of time [54]–[56]. One study concluded that snow cover depth increased across northern Eurasia (>60°N latitude), while a decrease occurred in southern Eurasia (<60°N latitude) suggesting that there has been an increase in precipitation and temperatures across the region related to surface climate warming in the Arctic region [56]. Another study examined recent changes of the onset date of green-up for portions of central Asia and determined that the steppe regions were highly influenced by spring precipitation [57]. A higher amount of precipitation in the spring has caused these regions to have earlier green-up dates than they had previously. Areas of the Mongolian steppe that had particular vegetation types and a higher level of spring soil moisture exhibited an overall trend of earlier green-up and an overall temperature increase was observed across much of the region as well as a warming trend at the beginning of the growing season. It is important to note that a significant part of interior Kazakhstan is primarily composed of the Kazakh steppe, which is an extension of the neighboring Mongolian steppe to the east. Because of the similarity and proximity of the steppe regions, the Kazakh steppe may also exhibit similar green-up patterns.

Other studies conducted at similar latitudes to Kazakhstan have examined the potential expansion and contraction of rangeland (i.e., grasslands used for the grazing of domestic cattle) and changes in phenological phases based on climate change [58], [59]. One study concluded that the northern latitudes of the US rangeland would experience an increase in growing season and an increase in plant production as well as an increase in peak standing crop [58]. An increase in forage across the northern latitudes resulted in less feed being needed to supplement the winter diet of cattle, potentially resulting in an increase in cattle numbers and an increase in calf weight [58]. Models used in this study predicted substantial variation in yearly green-up periods indicating an increasing sporadicity related to climate change. Overall, both plant and animal production increased for the northern latitudes according to the study. In addition to being more productive in most locations, rangelands also were predicted to expand into previously more arid locations. Changes in green-up and precipitation sporadicity in conjunction with rangeland expansion could indicate that some changes in the epidemiology of anthrax could occur such as longer anthrax seasons and an exposure of animals to more areas where *B. anthracis* may exist [36]. Because large anthrax epizootics often appear to occur after specific rain events (in association with overall hot, dry summer conditions [60], [61]), the increasingly sporadic rate of precipitation may also create some changes in the epidemiology of anthrax in the US as well as potentially in Kazakhstan. Changes in phenological phases that have occurred since the late 1930's were also studied, and maximal increases in earliness of photosynthetic activity were observed for latitudes between 45° N and 65° N [59]. While many plants did experience an overall increase in earliness of photosynthetic activity related to climate change, some plants were unaffected because they were more regulated by photoperiods [59].

Because anthrax remains a problem in livestock in the region and sometimes affects humans, further examination of the spatial ecology and geographic distribution of *B. anthracis* is imperative. Kazakhstan has limited veterinary services and predominantly rural agricultural practices, thus surveillance priorities should be dynamic and readily employed at any moment. The political boundary of Kazakhstan creates a larger amount of longitudinal change than latitudinal change and much of Kazakhstan lies within the upper mid-latitudes. Based on a previous study at similar latitudes [35] it is expected that there will be an overall contraction of *B. anthracis* environments by 2050 in the US with slightly more habitat contraction occurring in the southern latitudes.

The objective of this study is to determine the current and future potential geographic distributions of *B. anthracis* based on the Hadley Coupled Model version 3 climate predictions for 2045–2055 using multiple resolutions.

## Results

### Accuracy Metrics

Accuracy metrics were only performed on the models of current distribution because the location of future outbreak events is unknown and therefore unavailable for validation. The modeling processes for each of the three scenarios reached convergence of accuracy (0.01) prior to the maximum iteration setting of 1,000 models. The 55 km^2^ current scenario received an AUC score of 0.7045 and was significantly different from a line of no information (p<0.01). The model had a total omission of 0.0% and average omission of 5.5% meaning that 100.0% of the independent (testing) locality data were predicted correctly by at least one model and 94.5% of the independent locality data were predicted correctly by all models in the best subset. The 8 km^2^ current scenario received an AUC score of 0.6502 (p<0.01). The model had a total omission of 5.1% and average omission of 10.2%. The BioClim current scenario received an AUC score of 0.6995 (*p*<0.01). The model had a total omission of 5.1% and average omission of 10.0%. All accuracy metrics for the current predictions are summarized in Table 1.

**Table 1:** Accuracy Metrics for the current predicted distributions from each GARP experiment.

Metric	55 km^2^	8 km^2^	(BioClim)
*n* to build models	125†	218†	218†
*n* to test models	22	39	39
Total Omission	0.0	5.1	5.1
Average Omission	5.5	10.2	10.0
Total Commission	50.27	51.71	35.91
Average Commission	59.59	62.33	53.44
AUC*	0.70 (z = 7.7§, SE = 0.06)	0.65 (z = 9.8§, SE = 0.05)	0.69 (z = 9.0§, SE = 0.05)

* AUC  =  area under curve.

† *n* was divided into 50% training/50% testing at each model iteration.

§ p<0.001.

**Figure 1:**
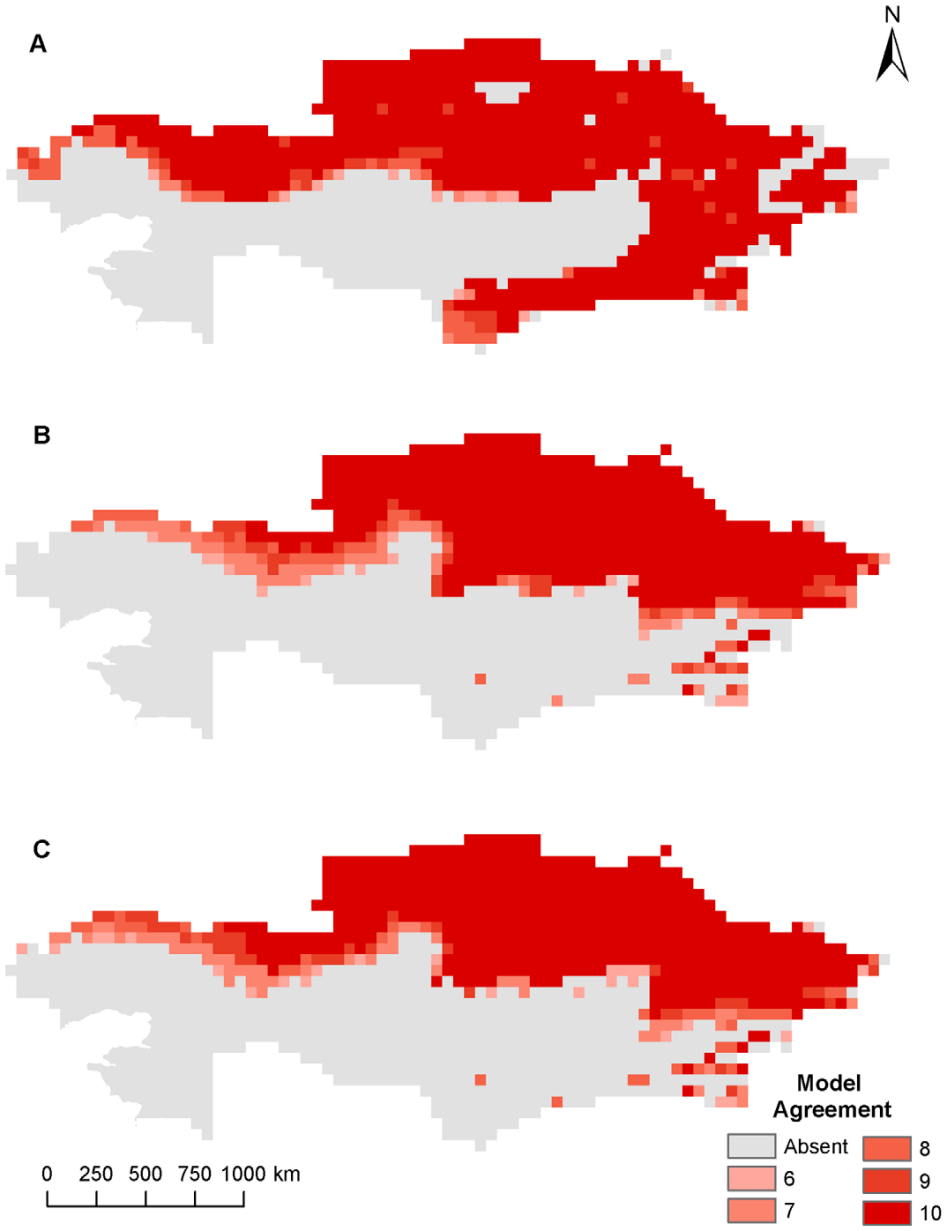
Current and future geographic distribution of *Bacillus anthracis* using climate data at 55 km^2^. (A) current geographic distribution, (B) A2 future climate scenario, (C) B2 future climate scenario. Color ramp indicates model agreement, with darker areas representing areas with high model agreement or greater confidence in the GARP prediction.

*Note: Independent data used for accuracy metrics appear in figure 1 (yellow points)*.

### Current and Future Distributions of *B. anthracis*

**Figure 2:**
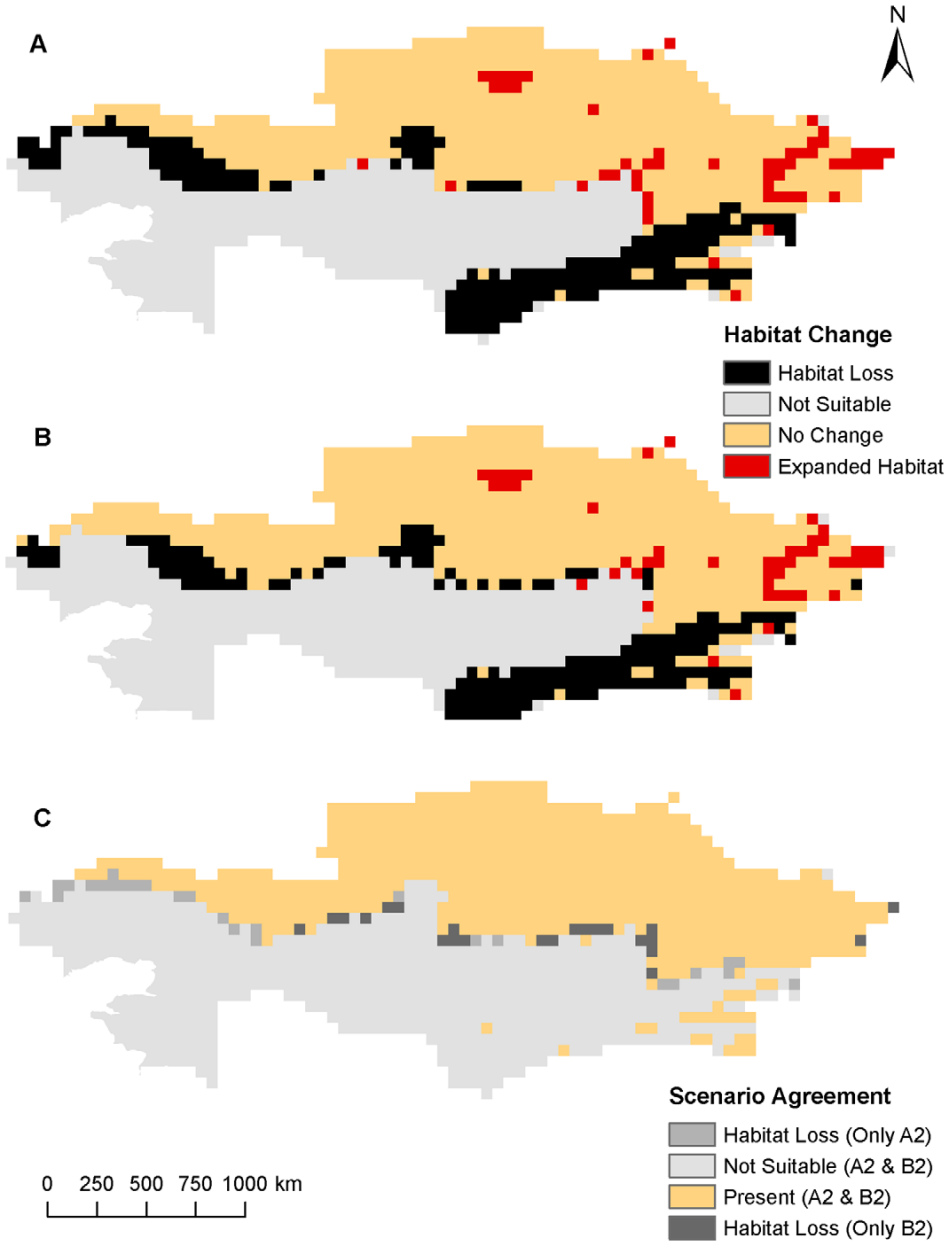
Comparison of predicted *B. anthracis* habitat changes from both climate scenarios using five variables at a resolution of 55 km^2^. Potential future habitat changes based on the A2 climate change scenario (A) and the B2 climate change scenario (B). Differences between each climate change scenario (C).

Current and future climate grid data were examined at the near-native resolution to verify if broad agreement occurred between 55 km^2^ outputs and the higher resolution 8 km^2^ climate data using non-bioclimatic variables. At the 55 km^2^ resolution areas of northern and southeastern Kazakhstan were predicted to be suitable for *B. anthracis* survival, while the A2 and B2 climate change scenarios predicted smaller geographic distributions in southeastern Kazakhstan as well as slightly smaller geographic distributions in interior and western Kazakhstan (Figure 1A–C). Overall the predicted current distribution of *B. anthracis* stretches across the northern tier, eastern quarter, and southeastern regions of Kazakhstan. It is predicted that these areas are potentially maintaining suitable environments for *B. anthracis*. The northern predictions follow a line of latitude approximately 48° N from West Kazakhstan to the eastern area of the Karaganda oblast near Lake Balkhash where the predictions then extend southward to the oblast of Aktobe. Model agreement decreases south of 48° N latitude in the southern half of the Karaganda oblast where no model predicts suitable habitat for *B. anthracis*. From eastern Karaganda oblast, habitat suitability expands farther to the south to encompass the eastern oblasts of Kazakhstan including nearly all of the Pavlodar, Almaty, and East Kazakhstan oblasts with slightly less suitability in the higher altitudes of the Altay Mountains in far eastern East Kazakhstan and the Tian Shan Mountains in the southern and southeastern regions of the Almaty oblast. The southern half of the Zhambyl and South Kazakhstan oblasts are also areas of high suitability with less model agreement in the north closer to their borders with the Karaganda oblasts and the Kazakh Steppe. Only the extreme southeastern areas of the Kyzylorda oblast provide potentially suitable habitat for *B. anthracis* while areas in the Kazakh Steppe and around both the Aral and Caspian Seas are not predicted to support *B. anthracis*. When considering the A2 and B2 climate change scenarios, a noticeable change occurs in many areas of Kazakhstan including parts of West Kazakhstan and Aktobe where a suitable environment for spore survival recedes to only the northern-most reaches of each oblast. While the southern half of Kostanay exhibits a contracting suitable environment, the northern half of the oblast and most of Akmola, North Kazakhstan, and Pavlodar, which border Siberian Russia, retain a suitable environment for *B. anthracis* spore survival. Contraction also occurs in the southern areas of Almaty, Zhambyl, and South Kazakhstan bordering Kyrgyzstan, China, and Uzbekistan. The predicted changes were more easily discernible in Figure 2A–C where areas of predicted habitat expansion and contraction were delineated for each climate change scenario and the percentages of habitat change were summarized in Table 2.

**Table 2:** A comparison of habitat change (%) between SRES A2 and B2 climate change scenarios for each GARP experiment.

Habitat Change	A2 Scenario (55 km^2^)	B2 Scenario (55 km^2^)	A2 Scenario (8 km^2^)	B2 Scenario (8 km^2^)	A2 Scenario (BioClim)	B2 Scenario (BioClim)
Expanded Habitat	4.15%	3.63%	0.71%	0.89%	0.20%	0.52%
No Change	43.85%	44.67%	40.94%	29.28%	36.81%	22.54%
Not Suitable	37.04%	37.56%	36.12%	35.94%	46.72%	46.76%
Habitat Loss	14.96%	14.15%	22.22%	33.88%	16.27%	30.18%

**Figure 3:**
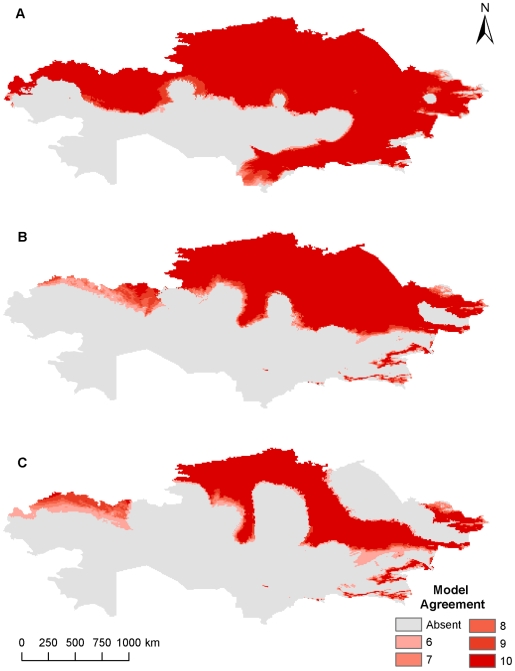
Current and future geographic distribution of *Bacillus anthracis* using climate data at 8 km^2^. (A) current geographic distribution, (B) A2 future climate scenario, (C) B2 future climate scenario. Color ramp indicates model agreement, with darker areas representing areas with high model agreement or greater confidence in the GARP prediction.

**Figure 4:**
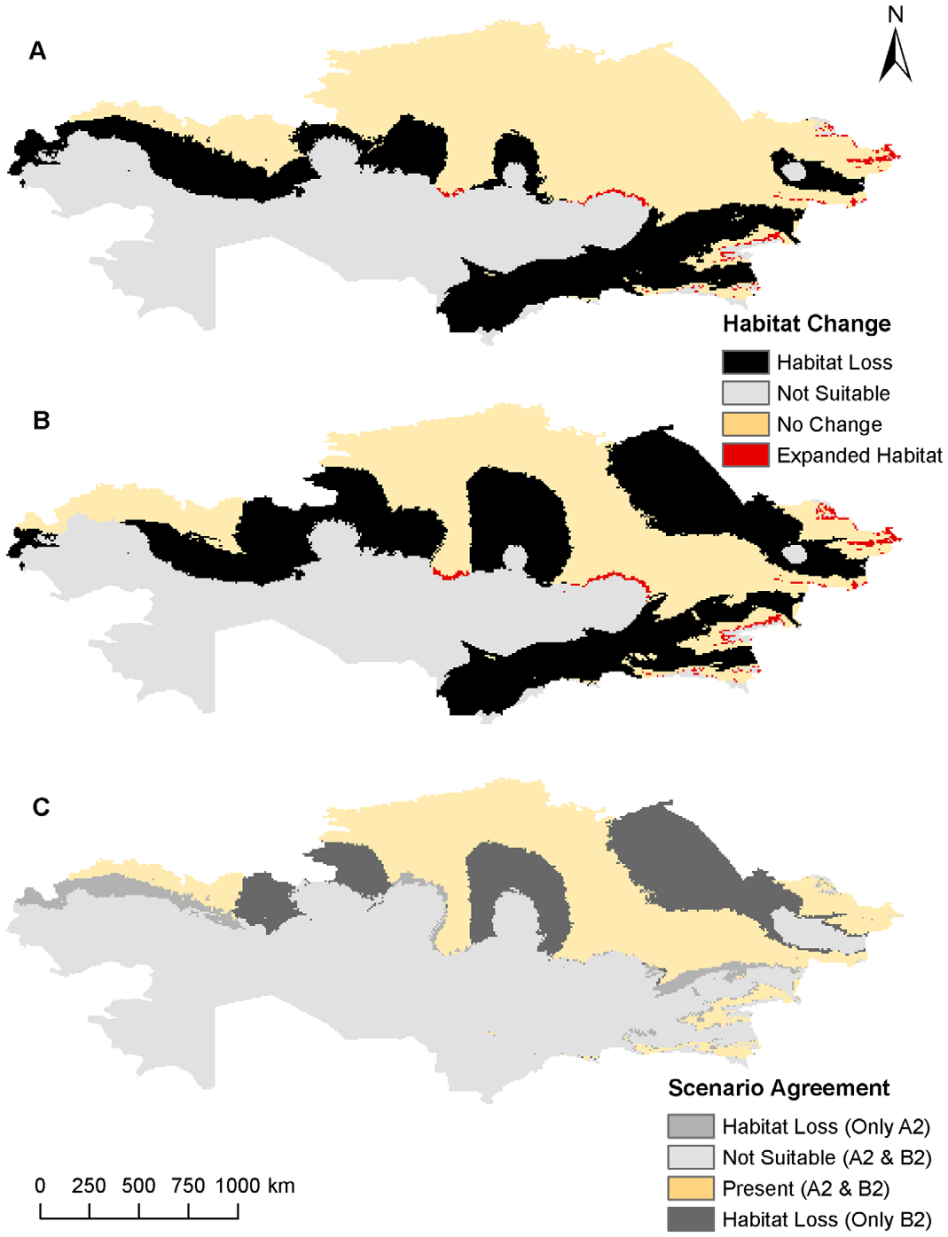
Comparison of predicted *B. anthracis* habitat changes from both climate scenarios using five variables at a resolution of 8 km^2^. Potential future habitat changes based on the A2 climate change scenario (A) and the B2 climate change scenario (B). Differences between each climate change scenario (C).

At the 8 km^2^ resolution areas of northern and southeastern Kazakhstan were predicted to be suitable for *B. anthracis* survival, while the A2 climate change scenario predicted a smaller geographic distribution in southeastern and eastern Kazakhstan and the B2 climate change scenario predicted a smaller geographic distribution in southeastern, northeastern, and central Kazakhstan (Figure 3A–C, Figure 4A–C). The models suggest that there are significant areas of southeastern and northwestern Kazakhstan where a suitable environment for *B. anthracis* will cease to exist, while most of the habitat will remain intact across the northern tier with marginal habitat losses closer to the interior of the country. Northeastern Kazakhstan may also experience drastic habitat loss, but only the B2 scenario predicts this response. The oblasts of West Kazakhstan, Aktobe, Almaty, Zhambyl, and South Kazakhstan could lose nearly all areas that were previously predicted to be suitable habitats for *B. anthracis* under current climatic conditions. There are also several very small areas of expanded habitat scattered across portions of interior and eastern Kazakhstan in Karaganda, East Kazakhstan, and Almaty. The percentages of expanded habitat, unchanged habitat, unsuitable habitat, and contracted habitat occurring across Kazakhstan for each climate change scenario at each resolution were summarized in Table 2.

**Figure 5:**
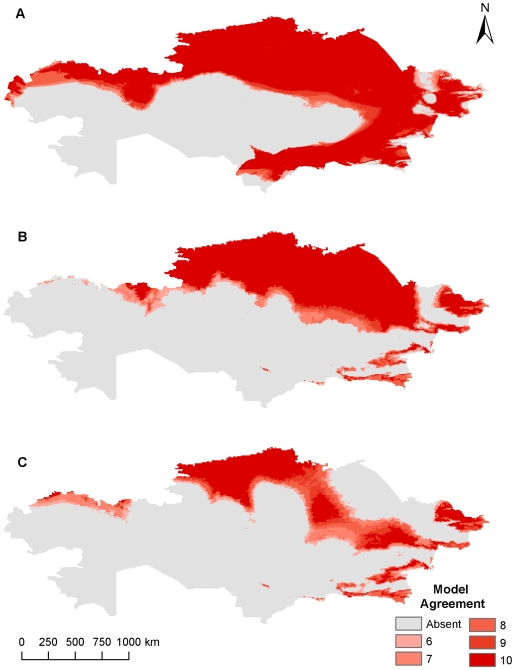
Current and future geographic distribution of *Bacillus anthracis* using BioClim variables at 8 km^2^. (A) current geographic distribution, (B) A2 future climate scenario, (C) B2 future climate scenario. Color ramp indicates model agreement, with darker areas representing areas with high model agreement or greater confidence in the GARP prediction.

**Figure 6:**
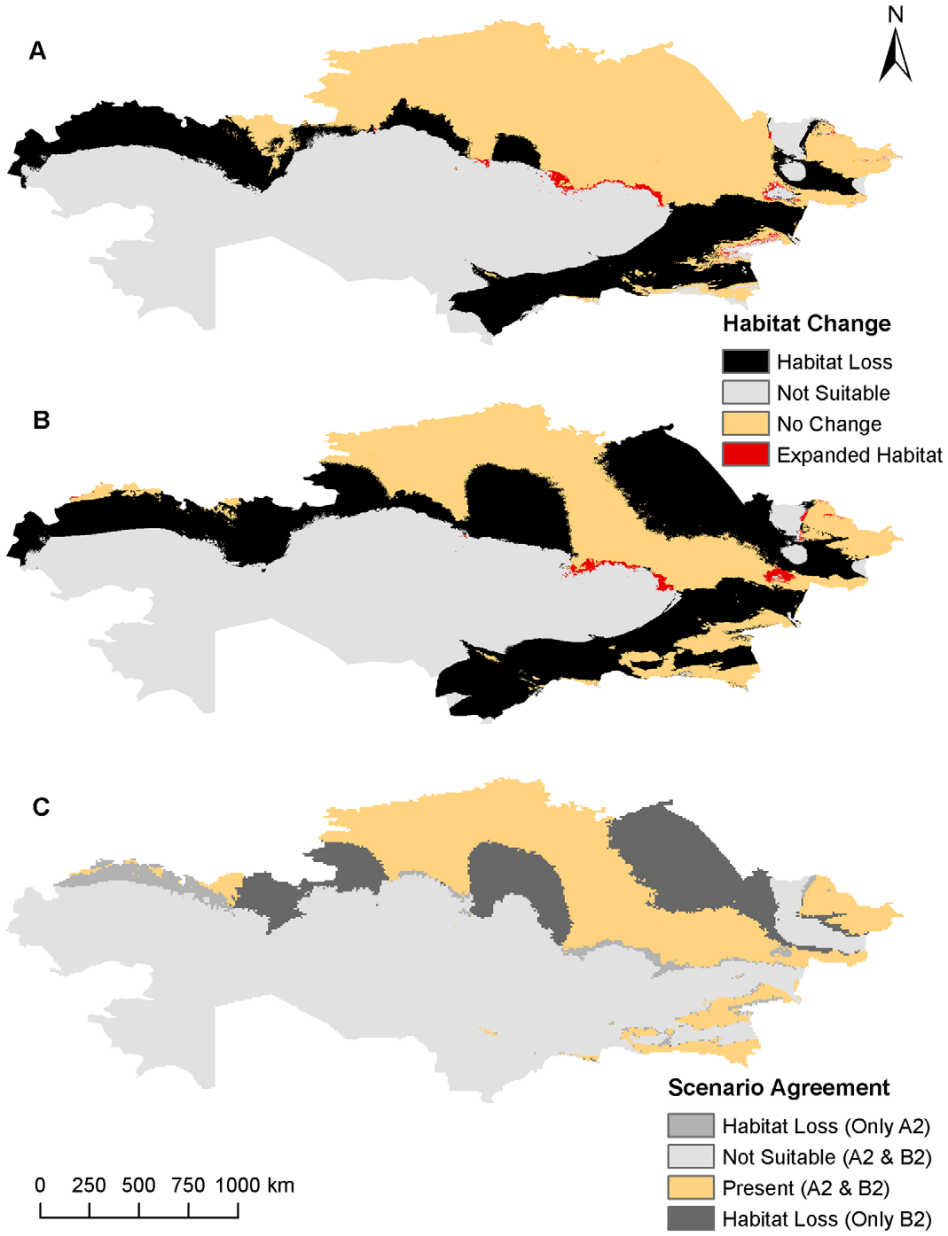
Comparison of predicted *B. anthracis* habitat changes from both climate scenarios using BioClim variables at 8 km^2^. Potential future habitat changes based on the A2 climate change scenario (A) and the B2 climate change scenario (B). Differences between each climate change scenario (C).

BioClim predictions are illustrated in Figure 5A–C and Figure 6A–C. Areas of northern and southeastern Kazakhstan were predicted to be currently suitable for *B. anthracis* survival, while the A2 climate change scenario predicted a smaller geographic distribution in southeastern and eastern Kazakhstan and the B2 climate change scenario predicted a smaller geographic distribution in southeastern, northeastern, and central Kazakhstan. The environmental parameters that allow for *B. anthracis* survival occur in only the northern-most section of West Kazakhstan and Aktobe in 2050 according to the B2 climate change scenario. Much of Akmola, Pavlodar, and East Kazakhstan are predicted to no longer maintain environments suitable for *B. anthracis*. A smaller geographic distribution is also predicted for the southeastern oblasts of Kazakhstan. The environments of interior Kazakhstan remain unsuitable for *B. anthracis* under the B2 scenario.

## Discussion

The accuracy metrics for the current scenarios confirms that GARP successfully predicted actual outbreak locations withheld from the model-building process. Very low total and average omission scores indicate a high predictive accuracy for each best subset presented. Additionally, an evaluation of individual test locations that were omitted in any of the current modeling scenarios shows that at least some of those are in areas unlikely to support *B. anthracis* in soils anyway based on the low frequency of such cases in a rather extensive time series of anthrax outbreaks. AUC scores were also reasonable for each scenario suggesting that our models are significantly better than random at identifying *B. anthracis* environments. As AUC directly reflects the relationship between omission and commission rates in its calculation [62], the 55 km^2^ scenario performed best of all in this study. While the BioClim experiment had a higher AUC than the 8 km^2^ experiment, it also predicted a smaller geographic extent of presence, so we would expect the AUC score to be higher. Given that both had equal total and average omission rates, it is unrealistic to consider any significant difference in performance of these two scenarios overall. While future changes in the distribution of *B. anthracis* are purely speculative, current models appear to be accurate regardless of resolution and climate datasets for 2050 show a broad level of overall agreement with habitat expansion in the north and contraction in the south. From this, it is arguable that *B. anthracis* has established a natural ecology across many regions of Kazakhstan, primarily the northern half, eastern quarter, and southeastern regions along the borders with Uzbekistan, Kyrgyzstan, and China. South-central and southeast regions of Kazakhstan that are now considered suitable environments for *B. anthracis* (and where a significant group of anthrax outbreaks have occurred over the past 70+ years [10]) may no longer have environmental conditions that support the long-term survival of *B. anthracis* according to projections from the A2 and B2 climate change scenarios at either spatial resolution.

A comparison between 55 km^2^ and 8 km^2^ climate data found that there was broad agreement across modeling experiments for the northern regions of Kazakhstan for the A2 climate change scenario. The southern areas of the Almaty, Zhambyl, and South Kazakhstan oblasts were predicted to experience drastic habitat loss (i.e., near total) at both resolutions, but drastic habitat loss in northern Kazakhstan was only predicted by the B2 climate change scenario at a resolution of 8 km^2^ (both 8 km^2^ and BioClim). The actual reasons for major differences in the predicted distribution by each resolution are uncertain, but a lack of data points, a relatively steep change in elevation, the calculation of bioclimatic variables, and/or the splining technique used to downscale WORLDCLIM data may be possible explanations. There is still some measure of uncertainty in future climate predictions even at crude resolution and all future estimates should be regarded with caution. More guidance from climatologists in selecting climate datasets is probably warranted when considering how various climatic or bioclimatic variables may affect the potential distribution of a species.

Currently, much anthrax surveillance is focused on the south-central and southeast regions of Kazakhstan because many anthrax cases have occurred there in an area of high human population density, i.e. observation bias. Based on future bioclimatic data alone there may be a reduction in anthrax cases reported for this region. Future changes in temperature and precipitation may also cause geographic contraction of rangeland in the southern regions where livestock currently graze, while causing geographic expansion of rangeland in the northern regions. This would subsequently allow more animals to graze in environments that are predicted to be suitable for *B. anthracis* in the north, while less grazing in the south in conjunction with a less suitable environment for *B. anthracis* may also lead to further reduction in epizootics for this region. While climatic conditions may have changed between 1960 and 2000, current climatic variables were based on weather conditions recorded between 1950 and 2000 thus we assume that locality data collected over the past several decades accurately reflect environmental parameters needed for *B. anthracis* presence on the landscape.

Overall, the hypothesis of predicted habitat loss in the south, but gain in the north was partially disproven. While a very small area of expanded habitat was consistently predicted in the northeastern regions of Kazakhstan, habitat loss was predicted in nearly every part of the country except the extreme northern regions bordering Russia (Figures 2C, 4C, 6C, Table 2). There was far more predicted habitat contraction in the southern regions of Kazakhstan than anticipated. Projected changes may reflect over-predictions of future habitat loss due to a lack of soils data, but nonetheless the southeast region should expect to observe some reduction in *B. anthracis* habitat.

The results of this current study agree with the results of similar continental scale studies where southern habitat reduction was also predicted due to the potential effects of climate change on other bacterial zoonoses [35], [43], [63] and we have documented this pattern in all three climate datasets used at both 55 km^2^ and 8 km^2^ resolutions. In the US, parts of the southern range of *B. anthracis* were predicted to contract by 2050, while some parts of the northern range were predicted to expand [35]. Nakazawa et al. [63] investigated the effects of climate change on tularemia and plague in the US with ENM and multiple climate change scenarios and predicted similar trends with more contraction occurring in the southern habitats than in the northern habitats for 2050. Similarly, a recent study that modeled the future distribution of plague-carrying ground squirrels in California using 1 km^2^ BioClim variables suggested a subtle geographic shift to higher latitudes and altitudes with a limited reduction at lower latitudes [43]. Collectively, these trends were not as drastic as the trends predicted for Kazakhstan, but contraction of a southern range was suggested for all three diseases. The more extreme changes in predicted distribution for Kazakhstan may be a result of the region potentially experiencing a more severe climatic change between now and 2050. However, it is not implausible that variables, such as soil conditions that were unavailable for this study, might limit the habitat reduction to smaller portions of the Kazakh landscape.

Research over the past several decades has indicated that sporadic vegetation growth occurred from year to year based on rainfall amounts in the desert and steppe regions of Kazakhstan [12]. This may infer that an increase in rainfall variability (as predicted in the region of central Asia by climate change scenarios) from year to year in desert and semi-arid steppe climates could equate to a more sporadic occurrence of anthrax outbreaks. While models may have predicted a complete disappearance of habitat for *B. anthracis* in certain regions, anthrax outbreaks may simply become increasingly sporadic, but not disappear altogether in these regions as the A2 and B2 climate change scenarios suggested. Changes in the landscape could limit (if desertification occurs) or increase (if an increase in rangeland occurs) the ability for cattle to migrate [12]. These potential changes in migratory patterns could help to spread or limit the range of anthrax outbreaks and subsequent *B. anthracis* introduction and survival. Cattle migration is already confined because of limitations placed on nomadic herdsmen over the past century [13]. Overall, cattle now graze on smaller areas than they did previously [12] and in areas where outbreaks have occurred, we would expect a possible increase in outbreak potential if population densities are high [64].

The current spatial distribution of *B. anthracis* follows similar latitudinal patterns as those predicted by a study in the United States with larger areas of the northern regions predicted to be endemic for *B. anthracis* compared to smaller areas predicted to be endemic for *B. anthracis* in the southern region [8]. This also closely follows the predicted current distribution of *B. anthracis* on the landscape of Kazakhstan (Aikembayev unpublished manuscript). The predicted areas of southern Kazakhstan traverse the foothills and mountain ranges of the Tian Shan and Altay Mountains, which have climates that are somewhat comparable to climates farther north. In maps of the projected distribution, it can also be determined that the suitable environments for *B. anthracis* (specifically in the southern regions) may move to areas of higher elevation greatly limiting its dispersal based on cattle grazing limitations [12]. Sheep, however, may not have similar grazing limitations because they are often transported either by foot or by truck/train to summer grazing areas in more mountainous regions [65]. Because of their mobility, sheep may be able to adapt to climate changes in the south more so than cattle and may subsequently remain in environments that continue to be suitable for *B. anthracis*. Rainfall has dictated livestock numbers and migratory patterns over the past several decades so this could in turn limit the contact that cattle may have with an environment where *B. anthracis* exist in the soil. The opposite may also be true if rainfall increases across many parts of Kazakhstan, more land could be available for grazing (similar to increases in forage in the northern latitudes of the United States [58]) thus allowing livestock to possibly move to more areas where they could come in contact with *B. anthracis*. An inverse relationship could potentially be created based on rainfall estimates that allow for livestock range expansion and *B. anthracis* range contraction. It is also important to consider the differences between the climate of Kazakhstan (continental with minimal influence from oceans) and the climate of the United States (surrounded by the Atlantic and Pacific Oceans as well as the Gulf of Mexico) when comparing the distribution of *B. anthracis* across the landscape of each.

Potential changes in seasonal vegetation patterns should also be examined in conjunction with typical seasonal patterns of anthrax outbreaks to determine if these patterns may coincide. Anthrax has a distinct seasonality and is primarily a summertime (May–October in northern latitudes) disease in both wild and domestic ruminants that is usually associated with wet springs and hot, dry summers followed by a rain event [66], [67]. The predicted rise in temperatures and potential for increasingly sporadic rain events across much of central Asia [48] could lead to spatial and temporal changes in where and when anthrax outbreaks occur in Kazakhstan. Rangeland expansion and contraction as well as changes in rangeland production in Kazakhstan could lead to a higher population of livestock in the northern regions, where *B. anthracis* is predicted to remain in 2050, and subsequently a potentially greater number of anthrax outbreaks. A rise in temperatures in the southern regions of Kazakhstan could create an environment that *B. anthracis* and/or livestock may not be able to survive in, thus potentially decreasing the number of anthrax outbreaks there. It has been shown in the US that areas supporting *B. anthracis* survival do overlap with livestock distributions, however they are not identical [35]. Livestock may graze in areas that are unsuitable for *B. anthracis* and likewise, *B. anthracis* may exist in areas that are either unsuitable or not used for livestock grazing.

It is also interesting to consider the possible evolutionary implications of these climate change scenarios. While the genetic understanding of *B. anthracis* in Kazakhstan is incomplete, recent efforts [10] have provided insights into the spatial distribution of Kazakh specific genotypes for the country. Employing the 8-primer MLVA-typing developed by Keim et al. [68], a recent study described 92 culture isolates from several historical outbreaks. The majority of these isolates belong to the A1.a genetic cluster and the majority of that diversity was located in the southern regions of Kazakhstan, predicted to no longer support *B. anthracis* in 2050 by all three modeling experiments and both climate scenarios. This might suggest that a reduction in suitable habitats in southern Kazakhstan may also correspond with a reduction in genetic diversity. It is difficult to estimate changes in diversity in the northern most extent of Kazakhstan, as no cultures were available for typing [10]. However, six of the 92 isolates from the existing data set represented a distinct member of the A3b sublineage. Interestingly, the 8 km^2^ and BioClim B2 scenarios suggest the northeastern region where these strains were isolated will no longer support *B. anthracis* in 2050.

When comparing climate change scenarios at a resolution of 8 km^2^, more habitat loss was predicted by the B2 climate change scenario–supposedly the more conservative (or optimistic) of the two scenarios. The B2 scenario delineates that more habitat loss may occur in the northern interior areas of Kazakhstan as well as the northeastern areas of Kazakhstan. Conversely, several small areas in southeastern and northwestern Kazakhstan that were classified as areas of habitat loss actually are predicted to retain their habitats in the B2 climate change scenario. While variations in the predicted precipitation and temperature changes for 2050 may have been the main reasons for distributional differences seen between the A2 and B2 scenarios, GARP used a combination of variables to create rule-sets that determined the environmental parameters that support *B. anthracis*. For example, a warmer and wetter environment in the north may create a more suitable environment for spore survival, but a warmer and drier environment in the south may also create a more suitable environment for spore survival in previously uninhabitable areas (e.g. in the higher elevations of the Tian Shan Mountains). Previous studies allude to the importance of examining specific rules within GARP rule-sets to evaluate changing relationships between variables across the landscape [8], [62] and variable combinations for this study should also be examined to further understand environmental constraints on the habitat of *B. anthracis*. Temperature and precipitation changes will not be uniform across the vast landscape of Kazakhstan. For this reason, the internal rule-sets need to be examined to determine which variables and combination of variables were most important in predicting the ecological niche of *B. anthracis*. A closer examination of individual variables and variable combinations derived through rule-sets may also help to reveal the potential driving mechanism(s) of the predicted habitat change for *B. anthracis* across many areas of Kazakhstan. Population growth and urbanization may also alter future predictions, but land cover use change may affect future predictions more if rangelands expand/contract in certain areas. Based on trends during the past century, Kazakhstan is not expected to experience drastic population growth or urbanization that would greatly modify future predictions.

## Materials and Methods

### Anthrax Occurrence Data

**Figure 7:**
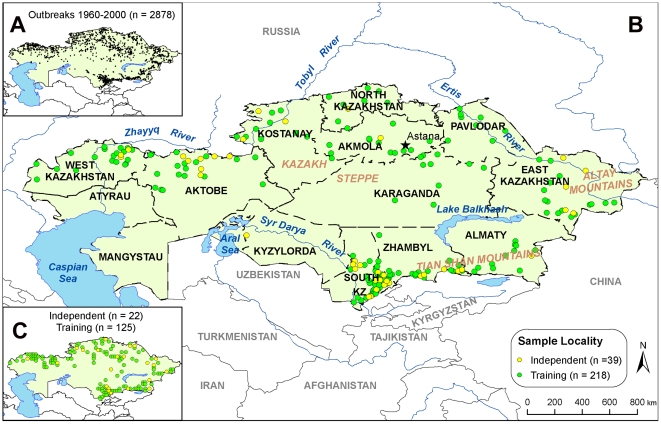
Map of Kazakhstan with anthrax locality data. Training data (green) were used to build models while independent data (yellow) were used to evaluate model accuracy. Inset A illustrates where all anthrax outbreaks occurred between 1960 and 2000. Inset B illustrates training and independent data used for building models at 8 km^2^ spatial resolution. Inset C illustrates training and independent data used for building models at a resolution of 55 km^2^.

A database totaling 3,947 outbreaks was constructed from historical records between 1937 and 2006 archived at the Kazakh Science Center for Quarantine and Zoonotic Disease (KSCQZD), Almaty, Kazakhstan. Of those, 3,929 records represented outbreaks in livestock. A total of 1,790 individual locations were reported, with 805 of those reporting repeat outbreaks^[10]^. Outbreak events in domesticated animals, large (cattle) and small (sheep and goats) ruminants, constituted the majority of the dataset. Following a previous ENM effort in Kazakhstan (Aikembayev unpublished manuscript), this study utilized data from 1960–2000 to most closely reflect the disease situation in the period after broad vaccination and control strategies had been introduced. A total of 1,181 outbreaks were reported in large ruminants and 1,303 outbreaks were reported in small ruminants across the database from 1960–2000 (Figure 7A).

A filtering technique was applied to these 2,484 outbreaks to create smaller datasets that contained only spatially unique points for each of two environmental data set pixel resolutions, 55 and 8 km^2^, respectively (Figure 7B–C). Points were considered spatially “unique” when they did not occur within the same pixel. GARP utilizes a single point per grid cell to identify it as present for *B. anthracis*. *Presence* and *absence* are the only two categories that GARP uses to separate grid cells and the presence of more than one point in a grid cell could create inflated accuracy metrics if points from the same grid cells are used to test whether or not GARP predicted a grid cell accurately. It would be the equivalent of using the same data for both the training and testing of a GARP model. Because GARP is a presence-only modeling approach, only species presence data are needed and pseudo-absences are generated from background areas where no species data occur [69].

### Current and Future Climate Datasets

There are four main emissions scenarios produced by the IPCC in its Special Report on Emissions Scenarios (SRES) and Third Assessment Report [45]. The first is the A1 scenario which accounts for a low population growth, but very rapid economic growth and globalization. Less focus is placed on sustainability and energy efficiency in this scenario. The second scenario is the B1 scenario which accounts for the same low population growth, but development that is more focused on environmental sustainability and accountability. The third is the A2 scenario and it estimates a very rapid population growth due to less convergence of fertility rates (approximately 15 billion by 2055) and only minor improvements in emission standards (increase of 1% of CO^2^) over that same time period. The fourth scenario is the B2 scenario which estimates a smaller global population growth than A2 (approximately 10 billion by 2055), but a higher population growth than both the A1 and B1 scenarios with more improvements in emission standards (increase of 0.5% of CO^2^) [45], [70]. We chose to use the HadCM3 (Hadley Coupled Model version 3) ensemble a versions of the A2 and B2 climate change scenarios for 2045–2055 (hereafter referred to as 2050) in order to evaluate the effects of both a conservative (B2) and a less conservative (A2) scenario of how climates may change over the next several decades. Other popular general circulation models (GCMs) such as the CGCM and CSIRO models use flux adjustments to offset and reduce significant climate drift, but it is most desirable to eliminate their use in the coupled models that we use for future climate simulations [71]–[73]. The HadCM3 model was chosen over other models because of its ability to produce a good simulation without the use of flux adjustments [74], [75].

Current and future climate grid data were freely downloadable (www.worldclim.org) on the WORLDCLIM website [76]. The initial interpolation of the grids was scaled to a relatively coarse resolution (∼111 km^2^) before a thin-plate smoothing spline algorithm was applied to reduce the surfaces to various finer resolutions that were validated against historical weather station data multiple times to reduce error associated with interpolation [76]. A resolution of 8 km^2^ was utilized for this study because village latitude and longitude coordinates were occasionally estimated to be greater than 1 km away from farms where anthrax outbreaks occurred. Current grids describing monthly precipitation values as well as maximum and minimum temperatures were available along with bioclimatic (BioClim) grids that were created through the manipulation of the aforementioned monthly variables in order to create more biologically meaningful variables that represent annual trends, seasonality, and extreme/limiting environmental factors [76]. One apparent advantage of the WORLDCLIM data set is the availability of BioClim variables which may be biologically more meaningful than annual mean, minimum, and maximum temperature and precipitation.

Future grids (e.g., for 2050 A2 and B2 climate change scenarios) describing monthly maximum and minimum temperatures and precipitation totals were also available, but bioclimatic grids were not available for future scenarios. For this reason, bioclimatic grids were calculated for both the A2 and B2 climate change scenarios. Bioclimatic variables were derived for current and future conditions following calculations provided on the WORLDCLIM website (www.worldclim.org). The calculations were performed with the use of the raster calculator within the Spatial Analyst extension of ArcMap 9.2 [77]. Once calculations were complete, a total of six world environmental variable grids were clipped to represent the spatial extent of Kazakhstan (Table 3). BioClim variables have been used in a recent study to develop current and future predictions of *Yersinia pestis* infected ground squirrels, *Spermophilus beecheyi*, in California using a similar approach to that described here [43].

**Table 3:** Environmental variables used for BioClim GARP models.

Environmental Variables	Name	Source
Annual Mean Temperature	BIO1	WorldClim (www. worldclim.org)
Temperature Annual Range	BIO7	WorldClim (www. worldclim.org)
Annual Precipitation	BIO12	WorldClim (www. worldclim.org)
Precipitation of Wettest Month	BIO13	WorldClim (www. worldclim.org)
Precipitation of Driest Month	BIO14	WorldClim (www. worldclim.org)
Elevation (Altitude)	ALT	WorldClim (www. worldclim.org)

Given that the native resolution of climate models is relatively crude, the accuracy of climate data resampled to a high spatial resolution is questionable [63]. To test for agreement between low and high resolution data sets, we constructed models using near-native resolution climate data directly from the IPCC at 55 km^2^. Without monthly data at low resolution, we did not calculate BioClim variables at 55 km^2^. To compare the resolution of 55 km^2^ and 8 km^2^, we used five variables to construct models at both resolutions: elevation, total annual precipitation, mean temperature, minimum annual temperature, and maximum annual temperature. A model using identical variables from the 8 km^2^ climate dataset was constructed in order to make a fair comparison between the two resolutions. Current and future climate grids were clipped and resampled to represent the spatial extent of Kazakhstan at these resolutions.

### Modeling Scenarios

For this study, we modeled the current geographic distribution of *B. anthracis* using three different scenarios at two different resolutions. The first two scenarios contained five environmental variables that described temperature, precipitation, and elevation that were used to create two models of the potential current distribution of *B. anthracis*. The first scenario utilized the five variables at a resolution of 55 km^2^ (herein referred to as 55 km^2^), while the second scenario utilized the five variables at a resolution of 8 km^2^ (herein referred to as 8 km^2^). The third scenario utilized six environmental variables that included elevation and five bioclimatic variables (herein referred to as BioClim; Table 3). Two models of the future distribution of *B. anthracis* were also created for each of the three scenarios. Temperature and precipitation trends predicted for 2050 by the *A2 climate change scenario* and *B2 climate change scenario* were used to construct the models and compare the future potential distributions to the current predicted distribution.

### Implementation and Methodology of Desktop GARP and Accuracy Metrics

The specific ENM chosen for this study was the Genetic Algorithm for Rule-set Prediction (GARP [69]). GARP is a presence-only genetic algorithm that models species' potential geographic distributions through an iterative process of training and testing that occurs through resampling and replacement of input data [69]. A pattern matching process is applied that finds non-random relationships between species localities and specific variables that describe the environment. These relationships are written as a series of if/then logic statements (known as rules) that define whether conditions within the rule are defining presence or absence. A GARP “model” is a combination of 50 rules that define the landscape as present or absent and the resulting rules are known as a rule-set. The rules consist of four specific types: range, negated range, atomic, and logistic regression [69]. GARP is genetic, meaning that rule development is done through an automated process, whereby rules are randomly generated, tested with internal statistical tests, and modified (through the rules of genetic evolution–point mutations, crossovers, deletions, insertions) [69] to determine which rules to keep and delete based on their accuracy at predicting internal testing data. Data splits occur both internally and externally for the purpose of model evaluation and are established by the user. A *best subset* of models is usually created during an experiment. A best subset is a group of a user-defined number of models from an experiment that meet omission and commission criteria established by the user as a means of selecting those models that best balance between low omission and median commission values [78].

While GARP has received some criticism as a “black box”[79], or being less precise than more recently developed tools [80], recent studies have shown GARP to perform well [8], [81] and it should be noted that this criticism was in part due to evaluations based on an unequal calculation of the accuracy metric used [82], [83]. Part of this confusion is also due to a conflation of ecological niche modeling and species distribution modeling [84]. Here we employ the former, while the criticism [80] was concerned with the latter.

Spatially unique point data were randomly split once into 85% training and 15% testing data subsets (Figure 7B–C) prior to model development using SPSS (version 16.0) [85]. The same 85% training datasets were used within the model-building process for all models, while the 15% testing datasets was withheld completely from the modeling experiments to evaluate the predictive accuracy of the models *post hoc*. Maps were then created from GARP outputs to identify the potential geographic distribution of *B. anthracis* based on the modeled niche definitions. Because GARP is a two-step process, first modeling in variable space and then projecting onto the landscape, it is plausible to project current rule-sets onto the potential future conditions of a landscape. This current study employed the Desktop GARP version 1.1.6 [DG] software application, an open source modeling program (http://www.nhm.ku.edu/desktopgarp/).

### Modeling Parameters

For all modeling scenarios, the training data were uploaded into DG with a 50/50 internal data split, meaning that 50% of the data were used within GARP to construct models and the remaining 50% were used for internal accuracy assessment of the rule-set and model building process. We employed 200 modeling runs using a convergence limit of .01 and 1000 max iterations using all four rule-types. The best subsets procedure was implemented to select optimal models for *B. anthracis* using an extrinsic omission measure and the selection of 20 models under a hard omission threshold of 10% and a commission threshold of 50%. This produces a 10-model best subset, where the 10 models with an accuracy of 90% or greater and closest to the median commission value are chosen to represent the potential geographic distribution. These 10 models were imported into ArcGIS and summated using the raster calculator routine in the Spatial Analyst extension. These maps represent values between 0 and 10, with 0 equally “absent” and values of 1 through 10 representing the number of models from the best subset that predicted that pixel as present; the greater the number, the higher the confidence in the model outcome [29]. Summated maps were produced for each modeling scenario in this study. A map of the current distribution and two maps of the projected distribution (i.e., A2 and B2 climate change scenarios) were created to show the potential geographic distribution in 2050 for each scenario.

The accuracy of the current distribution was then quantified through the use of accuracy metrics, which utilized the 15% testing data that was withheld from the modeling experiment. A receiver operating characteristic (ROC) analysis was used to produce area under the curve (AUC) scores. Additionally, two measures of omission (i.e., total and average), and two measures of commission (i.e., total and average) were also calculated for the current distribution model output. An AUC score ranges from 0.5 (lowest predictive accuracy – completely random) to 1.0 (perfect score–points were predicted 100% of the time), but AUC measurements are not ideal for validating the accuracy of GARP because they are subject to an area effect [21], [62], [83]. GARP usually only makes predictions across a small portion of the ROC plot, but AUC scores are measured across the entire area, not just the area predicted by GARP [83]. Because of this, ROC measurements should be regarded with caution. A recent study noted that the relative poorness of AUC scores is not necessarily a failure of GARP to predict an accurate distribution, but rather limitations of the statistics that are currently used to test model accuracy [62]. To provide a more robust evaluation of the models we presented AUC scores but along with measures of omission and commission that were based on the 15% testing subset [62].

### Analysis of Habitat Change

Summated maps from the best subset were reclassified to visualize the habitat changes between the current predicted distribution and the A2 and B2 scenarios. Grids for the current distribution and the projected A2 and B2 distributions were reclassified as presence (6 or more models agree) or absence (5 or fewer models agree). The raster calculator was then used to subtract the projected distributions from the current distribution. In total, two maps were produced representing habitat change (i.e., habitat expansion, habitat loss, no habitat change, unsuitable environment) occurring for the A2 and B2 climate change scenarios at each resolution and modeling scenario. The percentages of area occupied for each of the four categories of habitat change were tabulated.
